# Achondroplasia management in the era of targeted therapies: a meta-analysis of C-type natriuretic peptide analogs

**DOI:** 10.1210/jendso/bvag121

**Published:** 2026-06-16

**Authors:** Abul Bashar Mohammad Kamrul-Hasan, Suma Uday, Lakshmi Nagendra, Deep Dutta, Saptarshi Bhattacharya, Joseph M Pappachan, Ambika P Ashraf

**Affiliations:** Department of Endocrinology, Mymensingh Medical College, Mymensingh 2200, Bangladesh; Department of Pediatric Endocrinology, Diabetes & Metabolic Bone Disease, Narayana Health City, Bengaluru 560099, India; Department of Metabolism System Science, University of Birmingham, Birmingham B15 2TT, UK; Department of Endocrinology, JSS Medical College, JSS Academy of Higher Education and Research, Mysuru, Karnataka 570015, India; Department of Endocrinology, CEDAR Superspeciality Healthcare, New Delhi 110075, India; Department of Endocrinology, Indraprastha Apollo Hospitals, New Delhi 110076, India; Faculty of Science, Manchester Metropolitan University, Manchester, Greater Manchester M15 6BH, UK; Department of Endocrinology, Countess of Chester Hospital NHS Foundation Trust, Chester, Cheshire CH2 1UL, UK; Department of Endocrinology, Kasturba Medical College, Manipal, Manipal Academy of Higher Education, Manipal 576104, India; Division of Pediatric Endocrinology and Diabetes, University of Alabama at Birmingham, Birmingham, AL 35243, USA

**Keywords:** achondroplasia, short stature, skeletal dysplasia, C-type natriuretic peptide, *FGFR3*, vosoritide, navepegritide

## Abstract

**Context:**

No prior meta-analysis has systematically assessed efficacy and safety of C-type natriuretic peptide (CNP) analogs within the context of evolving understanding of FGFR3 biology and achondroplasia natural history.

**Objective:**

To evaluate the safety and efficacy of CNP analogs in children with achondroplasia and contextualize clinical outcomes and natural history.

**Methods:**

Systematic review of randomized control trials and real-world studies evaluating the safety and efficacy of CNP analogs (vosoritide and navepegritide) in children aged <18 years with genetically confirmed achondroplasia was performed. Coprimary outcomes of interest were adverse events (AEs) and changes from baseline in annualized growth velocity (AGV) at the end of the trials. Secondary outcomes included changes from baseline in height *Z*-score, standing height, and upper-to-lower body segment (ULS) ratio.

**Results:**

Eleven studies (*N* = 542) were included, of which 4 RCTs (*n* = 326) with low overall risk of bias were meta-analyzed. Overall and serious AE rates were comparable between CNP analogs and placebo, except for higher relative risks of injection site reactions (1.65), urticaria (4.04), and swelling (3.57). C-type natriuretic peptide analogs significantly increased mean differences in AGV (1.36 cm/year; 95% CI: 1.05-1.68; *P* < .00001) and standing height (1.24 cm; 95% CI: 0.47-2.01; *P* = .002), without short-term effect on ULS ratio. Real-world studies demonstrated sustained growth benefits with infrequent serious AEs or treatment discontinuations.

**Conclusion:**

C-type natriuretic peptide analogs provide slight but statistically meaningful improvements in linear growth in children with achondroplasia with acceptable short-term safety profile. Long-term studies are needed to define optimal timing of therapy on adult height, functional outcomes, and achondroplasia-related complications.

The therapeutic landscape for achondroplasia (ACH) is rapidly evolving. In addition to C-type natriuretic peptide (CNP) analogs, mechanism-based investigational approaches include direct inhibition of fibroblast growth factor receptor 3 gene (*FGFR3*), ligand traps, downstream pathway modulators, and longer-term gene-based or growth plate–targeted strategies [1]. In this context, rigorous synthesis of randomized trial (RCT) data for CNP analogs, the first approved disease-modifying therapies, integrated with contemporary understanding of *FGFR3* biology and natural history, is essential for benchmarking current therapies and guiding future innovation. In this integrative review, we provide a comprehensive overview of the pathophysiology and natural history of ACH and combine this framework with a quantitative meta-analysis of RCTs evaluating CNP analogs (vosoritide and navepegritide). By contextualizing pooled efficacy estimates within the biologic and clinical realities of ACH, we aim to inform the current state of disease-modifying therapy with both quantitative efficacy estimates and a broader understanding of disease progression and therapeutic targets.

## Epidemiology and pathophysiology

Gain-of-function mutations of FGFR3 are the most common genetic cause of disproportionate short stature, of which ACH (usually associated with a point mutation at FGFR3 c.1138) describes the more severe phenotype. Achondroplasia has an estimated incidence of 1 in 20 000 to 30 000 live births worldwide [2]. The prevalence varies by region but typically ranges from 1 to 9 per 100 000 people worldwide. European rates are consistently around 3.7 per 100 000 births, whereas some areas, such as North Africa/Middle East (34.3 per 100 000) and Sub-Saharan Africa (12.6 per 100 000), experience higher rates [3]. It results from a recurrent gain-of-function mutation in the *FGFR3* gene, usually a point mutation at nucleotide c.1138 (c.1138G>A or c.1138G>C), which leads to the substitution of arginine for glycine at codon 380 (p.Gly380Arg) [4, 5]. Although genetically simple, ACH is biologically complex, resulting in a lifelong disorder of endochondral ossification with multisystem clinical consequences. Under normal physiological conditions, *FGFR3* signaling acts as a regulatory “brake” on endochondral bone growth, modulating chondrocyte proliferation and differentiation within the growth plate [6, 7]. In ACH, constitutive activation of *FGFR3* amplifies this inhibitory signal, disrupting the balance between proliferation and hypertrophic differentiation of chondrocytes. This results in impaired longitudinal bone growth, premature closure of synchondroses, and characteristic skeletal dysplasia [6-8]. At the molecular level, overactive *FGFR3* signaling leads to persistent activation of downstream pathways, particularly the *RAS–MAPK/ERK* and *STAT1* cascades. Hyperactivation of *MAPK/ERK* signaling induces cell cycle arrest and premature differentiation of growth plate chondrocytes, thereby impairing endochondral ossification. Experimental models with constitutive activation of *MAPK/ERK* signaling in chondrocytes closely recapitulate the ACH phenotype, underscoring the central role of this pathway in growth plate failure [8-10]. Clinically, this manifests as shortened long bones with rhizomelic limb disproportion, macrocephaly with frontal bossing, and narrowing of the skull base and spinal canal [2].

## Natural history

The natural history of ACH is characterized by growth failure that begins prenatally and progresses throughout childhood [2]. Longitudinal cohort studies, including the CLARITY natural history study, have established ACH-specific growth curves that demonstrate a progressive decline in height SD scores (SDSs) relative to average-stature peers [11]. The most pronounced deficit occurs in the proximal long bones, resulting in persistent rhizomelic disproportion and altered upper-to-lower body segment (ULS) ratios. Without intervention, the mean adult height is approximately 131 cm in men and 124 cm in women [12]. The clinical burden of ACH extends far beyond stature. Impaired endochondral ossification at the skull base leads to premature fusion of synchondroses and foramen magnum stenosis, which can cause cervicomedullary compression, central apnea, and increased risk of sudden death in infancy [13, 14]. Progressive narrowing of the spinal canal with worsening thoracic kyphosis and lumbar lordosis contribute to spinal stenosis, chronic back pain, and neurogenic claudication later in life, frequently necessitating surgical decompression in adulthood [15]. Additional complications include obstructive sleep apnea due to midface hypoplasia and upper-airway crowding, recurrent otitis media with conductive hearing loss, and orthopedic manifestations such as genu varum, limited elbow extension, joint pain, and early osteoarthritis. Functional limitations in activities of daily living, including toileting, hygiene, and reaching, underscore the real-world impact of skeletal disproportion and contribute to reduced health-related quality of life [13, 16, 17].

## Targeted therapies

Against this backdrop of *FGFR3*-driven pathophysiology and its clinical sequelae, the elucidation of *FGFR3* signaling pathways has enabled the development of targeted, mechanism-based therapies. Among these, CNP has emerged as a critical counter-regulatory factor. CNP is produced naturally by proliferative and prehypertrophic chondrocytes. It binds to the natriuretic peptide receptor-B (NPR2/GC-B) on the chondrocyte surface, thereby triggering the production of cyclic guanosine monophosphate (cGMP) and activating protein kinase G [18, 19]. *CNP* signaling antagonizes *FGFR3*-mediated *MAPK/ERK* activation, in part through inhibition of *RAF-1*, thereby restoring chondrocyte proliferation and promoting more normal endochondral ossification. Importantly, this effect bypasses the mutant FGFR3 receptor itself and instead targets a shared downstream effector pathway. Conversely, *FGFR3* signaling can suppress NPR2 activity through dephosphorylation, highlighting a bidirectional regulatory relationship between these pathways [19, 20]. Despite its therapeutic promise, native CNP is unsuitable for clinical use because of rapid degradation by neutral endopeptidases and clearance via the Natriuretic Peptide Receptor C (NPR-C) receptor, resulting in a plasma half-life of only minutes. To overcome these limitations, long-acting CNP analogs have been engineered [21]. Vosoritide (BMN-111) is a 39-amino acid polypeptide engineered for enhanced stability compared with endogenous CNP. It includes a proline-glycine modification at its N terminus, which confers resistance to enzymatic degradation, thereby prolonging its biological activity and enabling once-daily subcutaneous administration [22]. Navepegritide (TransCon CNP) is a long-acting prodrug conjugated via a cleavable linker to a polyethylene glycol carrier molecule, designed to provide sustained systemic CNP levels upon weekly subcutaneous administration [23, 24]. Because vosoritide and navepegritide share a common upstream target (*NPR2/GC-B*) and converge on antagonism of *FGFR3*-mediated *MAPK/ERK* signaling, it is biologically coherent to evaluate them together as a therapeutic class. This shared mechanism provides a strong rationale for pooled analyses of CNP analogs and supports interpretation of clinical outcomes within a unified pathophysiologic framework.

The natural history of ACH directly guides the choice of clinically meaningful endpoints for disease-modifying therapies. Improvements in annualized growth velocity (AGV) and height SDS are important not merely as measures of stature, but because they may influence body proportionality, mechanical loading, and long-term risk of complications such as spinal stenosis and genu varum. Measures of body proportions, including ULS ratio and sitting height-to-standing height ratio, as well as patient-reported outcomes, provide critical insight into whether therapies promote more favorable skeletal geometry rather than simply increasing linear growth [25]. Most RCTs of vosoritide and navepegritide have therefore focused on AGV as the primary endpoint, with secondary outcomes including height SDS, body segment ratios, and safety measures such as injection site reactions and transient hypotension [26-29]. While individual trials have demonstrated increases in growth velocity with acceptable short-term safety profiles, in children with open epiphyses, uncertainties remain regarding age-dependent effects, durability of response, proportional skeletal changes, and long-term clinical impact. This systematic review and meta-analysis was therefore undertaken to synthesize current evidence base for the use of CNP analogs and to interpret these findings within the broader biological and clinical context of ACH.

## Materials and methods

The systematic review was conducted in accordance with the Cochrane Handbook for Systematic Reviews of Interventions and reported in accordance with the Preferred Reporting Items for Systematic Reviews and Meta-Analyses (PRISMA) checklist [30, 31]. This meta-analysis was prospectively registered in PROSPERO (CRD420251248977); the protocol summary is available online.

### Search strategy

A comprehensive search was performed across multiple databases and registries, including PubMed, Scopus, Web of Science, the Cochrane Central Register of Controlled Trials, and ClinicalTrials.gov. Google Scholar was also used to identify additional articles. The search covered these sources from their inception through December 5, 2025. Using the Boolean operators “AND” and “OR,” the following terms were searched: “achondroplasia,” “hypochondroplasia,” “FGFR3-related dysplasia,” “C-type natriuretic peptide analogue,” “CNP analogue,” “natriuretic peptide receptor 2 agonist,” “NPR2 agonist,” “vosoritide,” “BMN 111,” “BMN-111,” “navepegritide,” “TransCon CNP,” “ACP-015,” and “ASB20123.” The search used MeSH terms, document titles, abstracts, and keywords, without language restrictions, to identify published studies involving humans. Full search strategies are provided at the end of this manuscript (Table 1). The process also involved reviewing references cited in the articles collected for this research and relevant journals.

**Table 1:** The full search strategy and search results from different databases

PubMed	Scopus	Web of science	Cochrane CENTRAL
(“Achondroplasia”[Mesh] OR achondroplasia[tiab] OR hypochondroplasia[tiab] OR “FGFR3-related dysplasia”[tiab] OR “FGFR3 dysplasia”[tiab] OR “fibroblast growth factor receptor 3”[tiab]) AND (“Natriuretic Peptide, C-Type”[Mesh] OR “Natriuretic Peptide, C-Type”[tiab] OR “C-type natriuretic peptide”[tiab] OR CNP[tiab] OR “C-type natriuretic peptide analogue”[tiab] OR “CNP analogue”[tiab] OR “natriuretic peptide receptor 2 agonist”[tiab] OR “NPR2 agonist”[tiab] OR vosoritide[tiab] OR “BMN 111”[tiab] OR “BMN-111”[tiab] OR navepegritide[tiab] OR “TransCon CNP”[tiab] OR “ACP-015”[tiab] OR “ASB20123”[tiab]) AND (“humans”[Mesh] OR human*[tiab])	TITLE-ABS-KEY (achondroplasia OR hypochondroplasia OR “FGFR3-related dysplasia” OR “FGFR3 dysplasia” OR “fibroblast growth factor receptor 3”) AND TITLE-ABS-KEY (“C-type natriuretic peptide analogue” OR “CNP analogue” OR “natriuretic peptide receptor 2 agonist” OR “NPR2 agonist” OR vosoritide OR “BMN 111” OR “BMN-111” OR navepegritide OR “TransCon CNP” OR “ACP-015” OR “ASB20123” OR “C-type natriuretic peptide” OR CNP)	TS = ((achondroplasia OR hypochondroplasia OR “FGFR3-related dysplasia” OR “FGFR3 dysplasia” OR “fibroblast growth factor receptor 3”) AND (“C-type natriuretic peptide analogue” OR “CNP analogue” OR “natriuretic peptide receptor 2 agonist” OR “NPR2 agonist” OR vosoritide OR “BMN 111” OR “BMN-111” OR navepegritide OR “TransCon CNP” OR “ACP-015” OR “ASB20123” OR “C-type natriuretic peptide” OR CNP))	(achondroplasia OR hypochondroplasia OR “FGFR3-related dysplasia” OR “FGFR3 dysplasia”) AND (“C-type natriuretic peptide analogue” OR “CNP analogue” OR “natriuretic peptide receptor 2 agonist” OR “NPR2 agonist” OR vosoritide OR “BMN 111” OR “BMN-111” OR navepegritide OR “TransCon CNP” OR “ACP-015” OR “ASB20123”)

Abbreviation: CENTRAL, Cochrane Central Register of Controlled Trials.

### Study selection

The Population, Intervention, Comparison, Outcomes, and Study design (PICOS) framework served as the basis for establishing eligibility criteria for the clinical trials included in this study. The patient population (P) comprised children under 18 years old with genetically confirmed ACH. The intervention (I) involved subcutaneous injections of CNP analogs to treat ACH, whereas the comparison/control group (C), when present, received placebo injections. The outcomes (O) included the safety and tolerability of CNP analogs compared with placebo, as well as changes from baseline in AGV, height *Z*-score, ULS ratio, and standing height at the end of the trials. RCTs, single-arm open-label trials, or real-world studies (prospective or retrospective) lasting at least 6 months were considered eligible as study types (S) for inclusion. Placebo-controlled RCTs were included in the meta-analysis, while those without a placebo arm were included in the qualitative review. Studies involving animals, healthy humans, case reports, case series, conference proceedings, preprints, letters to the editor, extension phases of included trials, and articles lacking data on outcomes of interest were excluded. Three independent authors conducted the study selection process, first removing duplicates and then screening titles and abstracts against predefined inclusion and exclusion criteria. This step excluded irrelevant studies, leaving potentially eligible studies for further review. After reaching consensus, the authors retrieved the full texts of the remaining articles and independently assessed them for eligibility. Reasons for exclusion were documented for each article, as illustrated in the PRISMA flow diagram. Any disagreements were settled through consensus.

### Outcomes analyzed

The coprimary outcomes of interest were adverse events (AEs) and changes from baseline in AGV at the end of the trials. The secondary outcomes included changes from baseline in height *Z*-score, ULS ratio, and standing height at the end of the trials.

### Data extraction and dealing with missing data

Two authors independently extracted data using standardized forms. When several publications came from the same study group, their results were merged, and relevant data from each report were incorporated into the analysis. Patient characteristics, such as demographic information and comorbidities, from both included and excluded studies were documented in a table. The data extracted from all eligible studies for the review included first author, publication year, phase, trial registration number, country of study, key inclusion criteria, intervention details, sample size, percentage of female participants, mean age, baseline standing height, height *Z*-score, AGV, and follow-up duration. Data on both primary and secondary outcomes were also collected, as previously mentioned. For continuous outcomes, we extracted mean differences (MDs) and SDs between baseline and end-of-study values. The data were converted to MD (±SD) when reported in other formats, such as median or CIs, using the web-based Meta-Analysis Accelerator [32]. For categorical variables, we recorded the number of participants with the specific outcome and the total number of participants. The Supplementary Materials of the relevant studies were carefully reviewed. Relevant information was obtained via email from the corresponding authors and incorporated into the meta-analysis. Additionally, attrition rates—such as dropouts, follow-up losses, and withdrawals—were carefully examined. Any disagreements were resolved through consensus.

### Risk of bias assessment

Two authors independently evaluated the risk of bias (RoB). The Cochrane risk-of-bias tool for randomized trials version 2 (RoB2) was used to assess the RoB of the RCTs, while the Risk of Bias In Nonrandomized Studies of Interventions version 2 (ROBINS-I V2) was applied for nonrandomized intervention trials and retrospective cohort studies [33, 34]. Details of RoB2 and ROBINS-I V2 assessments are described elsewhere [35]. Disagreements were resolved through consensus.

### Statistical analysis

The Review Manager (RevMan) software, version 9.17.0, was used to conduct meta-analyses and generate forest plots [36]. The meta-analyses included forest plots combining data from at least 2 trials. The results were reported as risk ratios (RRs) for dichotomous variables and MDs for continuous variables, with 95% CIs. Random-effects models were selected to account for anticipated heterogeneity arising from variation in baseline population characteristics. The inverse variance method was consistently used. CIs were determined with the Wald-type approach, while Tau^2^ was estimated using the restricted maximum-likelihood method. A significance threshold of *P* < .05 was set.

### Assessment of and dealing with heterogeneity

The heterogeneity assessment started with examining the forest plots. Then, Chi-square tests were performed with N−1 degrees of freedom at the 0.05 significance level to determine statistical significance. The *I*^2^ test was also used in subsequent analysis; thresholds for *I*^2^ values were 25% for low heterogeneity, 50% for moderate heterogeneity, and 75% for high heterogeneity [37]. We conducted subgroup analyses of efficacy outcomes according to CNP analog (vosoritide vs navepegritide) and age group (<5 vs ≥5 years).

## Results

### Search results

**Figure 1:**
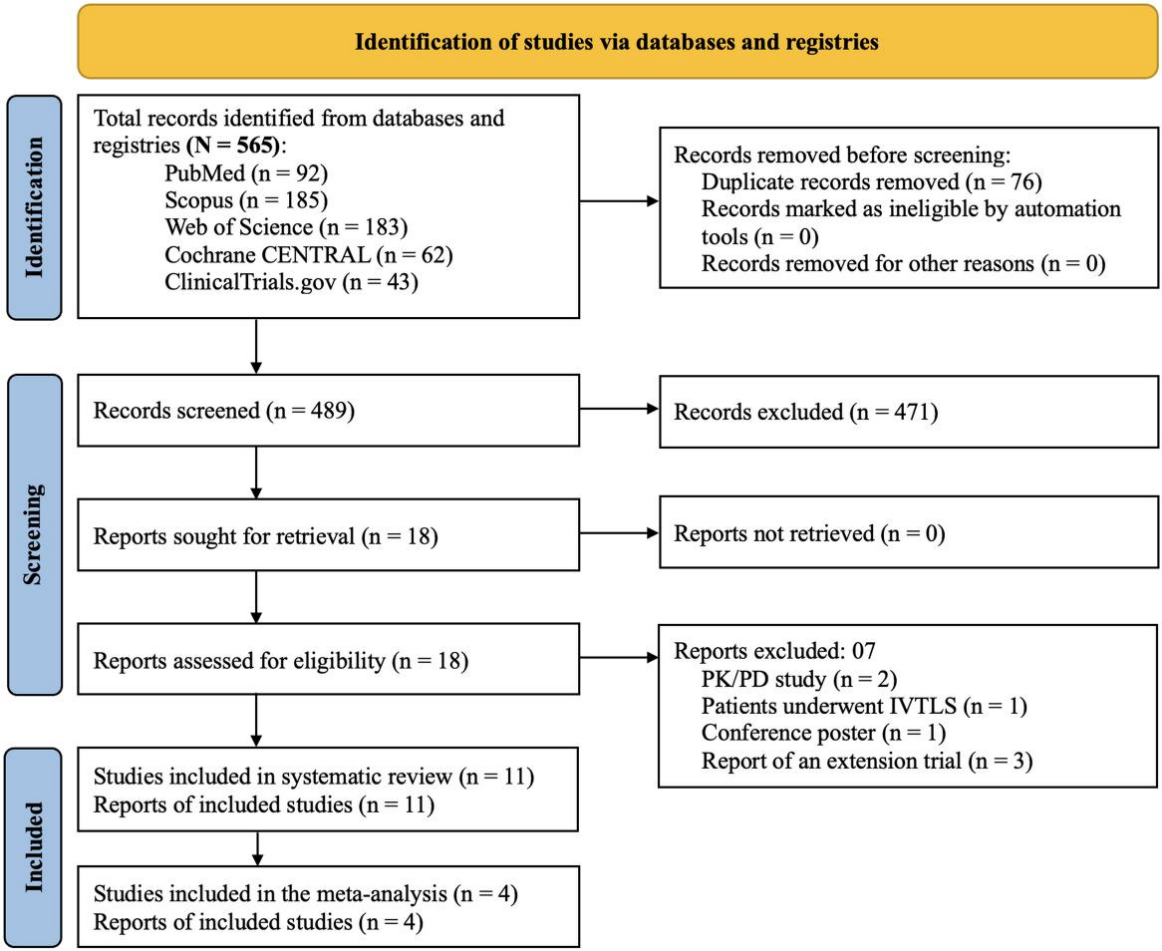
Flowchart on study retrieval and inclusion in the meta-analysis.

Figure 1 illustrates the study selection process, and Table 1 shows the full search strategy. The initial search identified 565 articles. After removing duplicates and screening titles, abstracts, and full texts, the number was narrowed down to 18. Finally, 11 studies (*N* = 542) that met all the predefined criteria were included in the systematic review [26-29, 38-44], of which 4 RCTs (*n* = 326) were included in the meta-analysis [26-29]. Seven studies were excluded [45-51], including 2 pharmacokinetic/pharmacodynamic studies [46, 47], 1 involving patients who underwent integrated vosoritide therapy with limb surgery [45], 1 conference paper [48], and 3 reports of the extension phases of included trials [49-51].

### Characteristics of the included studies

Table 2 summarizes the characteristics of the 4 placebo-controlled RCTs included in the meta-analysis. Of these, 1 was a phase 3 trial [26], and the other 3 were phase 2 trials [27-29]. Two RCTs involved vosoritide [26, 28], and 2 involved navepegritide [27, 29]. Two trials employed a single-arm intervention with vosoritide 15 μg/kg/day (Savarirayan et al [26]) and navepegritide 100 μg/kg/week (Savarirayan et al [29]). Savarirayan et al [27] evaluated 4 doses of navepegritide: 6, 20, 50, and 100 μg/kg/week. Savarirayan et al [28] divided the study subjects into 3 cohorts based on age at enrollment: cohort 1 (24-59 months), cohort 2 (6-23 months), and cohort 3 (<6 months). Vosoritide 15 μg/kg/day was used in cohort 1, while vosoritide 30 μg/kg/day was used in cohorts 2 and 3. All 4 RCTs had a 52-week duration. Baseline characteristics were statistically similar between the intervention and placebo groups across all included RCTs. Table 3 presents the details of studies [26-29, 38-44] included in the systematic review (qualitative synthesis) but excluded from the meta-analysis. Of these 7 studies, 2 were single-arm, open-label, phase 2 trials [39, 42], 1 was an open-label prospective study [43], and the remaining 4 were retrospective real-world studies [38, 40, 41, 44]. Vosoritide was used in all these studies at a dose ranging from 2.5 to 30 μg/kg/day. Savarirayan et al [42] enrolled 4 sequential cohorts to receive different doses of vosoritide (cohort 1: 2.5 μg/kg/day; cohort 2: 7.5 μg/kg/day; cohort 3: 15.0 μg/kg/day; cohort 4: 30.0 μg/kg/day). Shimada et al [44] had 2 groups: a growth hormone (GH)-naive group (Group A), receiving vosoritide alone, and a switch group (Group B), who transitioned from prior GH treatment. Other studies had only one group receiving vosoritide. Follow-up durations in these studies ranged from 1 to 6 years. Table 4 summarizes the studies excluded [45-51]; all used vosoritide.

**Table 2:** Summary of the placebo-controlled randomized controlled trials and baseline characteristics of the trial participants (*N* = 326)

Authors, Publication year [ref.], trial phase, registration, country	Major inclusion criteria	Trial arms		*N*	F (%)	Age (mean ± SD)	Standing height (cm) (mean ± SD)	Height *Z*-score (mean ± SD)	AGV (cm/year) (mean ± SD)	Upper-to-lower segment ratio (mean ± SD)	Trial duration
Savarirayan et al 2020 [26], Phase 3, NCT03197766, EudraCT 2015-003836-11, 24 sites in 7 countries	Age 5 to <18 years Ambulatory Genetically confirmed achondroplasia Completed at least 6 months of a lead-in, observational growth study	Vosoritide 15 μg/kg/day		60	48	8.35 ± 2.43 y	100.2 ± 11.9	−5.13 ± 1.11	4.26 ± 1.53	1.98 ± 0.2	52 weeks
		Placebo		61	46	9.06 ± 2.47 y	102.94 ± 10.98	−5.14 ± 1.07	4.06 ± 1.2	2.01 ± 0.21	
Savarirayan et al 2023 [27], Phase 2, (ACcomplisH), NCT04085523 and EudraCT 2019-002754-22, 16 sites in 8 countries	Age 2-10 years, prepubertal Ambulatory Genetically confirmed achondroplasia	Navepegritide 6 μg/kg/week		10	70	6.5 ± 2.6 y	90.63 ± 8.97	NA	NA	NA	52 weeks
		Navepegritide 20 μg/kg/week		11	27	6.3 ± 2.9 y	92.29 ± 12.1	NA	NA	NA	
		Navepegritide 50 μg/kg/week		10	30	5.2 ± 3.0 y	86.61 ± 12.97	NA	NA	NA	
		Navepegritide 100 μg/kg/week		11	55	5.8 ± 2.6 y	89.23 ± 12.82	NA	NA	NA	
		Placebo		15	33	5.9 ± 3.1 y	90.85 ± 14.92	NA	NA	NA	
Savarirayan et al 2024 [28], Phase 2, NCT03583697 and EudraCT 2016-003826-18, 16 sites in 4 countries	Age <60 months Genetically confirmed achondroplasia Completed a baseline growth study or observation period	Cohort 1 (24-59 months)	Vosoritide 15 μg/kg/day	15	53	39.6 ± 10.1 m	79.77 ± 4.87	−4.27 ± 0.81	4.74 ± 1.68	2.35 ± 0.17	52 weeks
			Placebo	16	56	44.3 ± 11.5 m	79.38 ± 6.79	−5.13 ± 1.15	4.2 ± 1.78	2.25 ± 0.19	
		Cohort 2 (6-23 months)	Vosoritide 30 μg/kg/day	8	38	17.0 ± 5.8 m	69.16 ± 5.6	−3.39 ± 0.84	11.51 ± 4.66	2.65 ± 0.3	
			Placebo	8	38	16.9 ± 6.2 m	66.45 ± 6.68	−4.21 ± 1.24	10.55 ± 4.78	2.68 ± 0.33	
		Cohort 3 (<6 months)	Vosoritide 30 μg/kg/day	9	44	5.6 ± 0.4 m	57.65 ± 0.64	−3.34 ± 1.02	21.19 ± 2.8	2.99 ± 0.47	
			Placebo	8	88	5.8 ± 0.6 m	58.15 ± 1.0	−2.65 ± 0.79	19.45 ± 7.55	2.87 ± 0.21	
Savarirayan et al 2025 [29], Phase 2b (APPROACH), NCT05598320, 10 sites in 7 countries	Age 2-11 years Genetically confirmed achondroplasia Naive to treatment with growth-promoting agents Height recorded at least 6 months prior to randomization	Navepegritide 100 μg/kg/week		57	45.6	NA	88.9 ± 12.9	−4.9 ± 1.0	4.0 ± 1.9	NA	52 weeks
		Placebo		27	48.1	NA	89.1 ± 11.5	−5.21 ± 0.9	3.8 ± 2.0	NA	

Abbreviations: AGV, annualized growth velocity; CNPA, C-type natriuretic peptide analogs; F, female; NA, not available.

**Table 3:** Summary of the nonplacebo-controlled trials and real-world observational studies with baseline characteristics of the participants

Authors, publication year, study place	Study type and major inclusion criteria	Study arms	*N*	Age (year), mean ± SD or range or median (IQR)	Height *Z*-score or SDS (mean ± SD)	AGV (cm/year) or AGV-SDS_ACH, mean ± SD or mean (CI)	Study duration
Cormier-Daire et al 2024 [38], France	Real-world, prospective study Age ≥5 years With achondroplasia and open epiphyses	Vosoritide 15 μg/kg/day	57	8.6 ± 2	−5.1 ± 1.04 (CDC)	NA	12 months
Dauber et al 2024 [39], USA	Single-arm, phase 2, open-label trial (NCT04219007) Prepubertal, age ≥3 years to <11 years for males and <10 years for females Standing height ≤−2.25 SDS on CDC growth chart	Vosoritide 15 μg/kg/day	24	5.86 ± 2.29	−3.28 ± 0.69 (CDC); −0.38 ± 0.76 (ACH-specific)	AGV: 5.12 ± 1.36	12 months
Reincke et al 2025 [40], Germany	Real-world, retrospective study Received vosoritide treatment for at least 12 months between October 2021 and May 2024	Vosoritide	34	Mean 7.52 (range 2.8-15.3)	−4.84 ± 1.0 (CDC); 0.37 ± 1.32 (ACH-specific)		12 months
Rua et al, 2025 [41], Portugal	Retrospective cohort study Treated with vosoritide for at least 6 months between January 2022 and June 2024	Vosoritide 15 μg/kg/day	27	7.3 ± 4.07	−5.08 ± 0.83 (WHO); −0.07 ± 1.13 (ACH-specific)	AGV: 4.25, (95% CI: 3.58-4.91)	24 months
Savarirayan et al 2019 [42], USA, Australia, France, UK	Phase 2, open-label, sequential-cohort, dose finding study (NCT02055157) Age 5-14 years, completed at least 6 months of a run-in observational growth study	Vosoritide 2.5-30 μg/kg/day	35	7.6 ± 1.7	−5.12 ± 1.026 (CDC)	AGV: 3.84 ± 1.625	42 months
Sawamura et al 2025 [43], Japan	Open-label, prospective study Age ≤15 years, had a minimum follow-up period of 1 year	Vosoritide 15 μg/kg/day	17	7.6 ± 2.7	−4.7 ± 0.9 (Japan); 0.2 ± 1.4 (ACH-specific)	NA	12 months
Shimada et al 2026 [44], Japan	Retrospective study Received vosoritide for at least 6 months between February 2018 and May 2025 Two groups: GH-naive group (Group A, *n* = 13), receiving vosoritide alone, and a switch group (Group B, *n* = 9), who transitioned from prior GH treatment	≥2 years: vosoritide 15 μg/kg/day; <2 years: vosoritide 30 μg/kg/day	22 (Group A 13; Group B 9)	Group A: 2.10 (0.37, 4.95); Group B: 10.1 (8.52, 11.3)	Group A: −4.56 (−5.36, −4.32) (Japan); −0.94 (−1.44, −0.34) (ACH-specific), Group B: −4.09 (−4.98, −3.10) (Japan); 0.99 (−0.39, 2.33) (ACH-specific)	AGV-SDS_ACH Group A: 0.95 ± 0.78; Group B: −0.54 ± 0.66	6 years

Abbreviations: ACH, achondroplasia; AGV, annualized growth velocity; CDC, US Centers for Disease Control and Prevention; CNPA, C-type natriuretic peptide analogs; F, female; NA, not available; SDS, SD scores.

**Table 4:** Summary of the excluded studies of vosoritide

Authors, publication year	Reason for exclusion	Study participants	Main outcomes
Allegri et al, 2025 [45]	Patients underwent IVTLS	Sample size: 16, age 12.6 ± 1.0 years 13 underwent limb lengthening surgery, and 3 underwent hemiepiphysiodesis surgery 15 started vosoritide between 6 months before and 6 months after the placement of an orthopedic device, 1 patient started vosoritide after 6 months	Treatment outcomes align closely with expectations No serious AEs ISR (85%), vomiting (27%), reduction in BP (13%)
Chan et al, 2022 [46]	PK and exposure–response study	PK of vosoritide and relationships between plasma exposure and efficacy, biomarkers, and safety endpoints were evaluated in a phase 2, open-label, dose-escalation study (*n* = 35 patients aged 5-14 years who received daily s.c. injections for 24 months) and a phase 3, double-blind, placebo-controlled study (*n* = 60 patients aged 5-18 years randomized to receive daily s.c. injections for 52 weeks)	The exposure–response relationships for changes in both annualized growth velocity, saturated at 15 μg/kg No evidence of accumulation with once-daily dosing
Galetaki et al, 2024 [47]	PK/PD study	Phase 2 trial of daily s.c. vosoritide 15 μg/kg/day in 24 prepubertal subjects with hypochondroplasia (12 females, mean age 5.9 ± 2.3 years, mean height −3.29 ± 0.68 SD) Plasma vosoritide levels were assayed using an electrochemiluminescence assay PD markers including serum CXM and urine cGMP production were measured at day 1, month 6, and month 12 visits	CXM levels increased from a baseline mean of 22.5 ± 6.5 to 41.6 ± 15.9 ng/mL after 12 months of treatment (*P* < .0001) Urine cGMP increased within 1 hour and peaked at 2 hours after injection The mean AUC for cGMP production was not significantly different at each study visit The maximum change in cGMP AUC correlated with PK AUC (*r* = 0.46, *P* = .0001) However, drug exposure, as measured by average PK AUC, did not correlate with any growth outcome CXM levels correlated with the prior 6-month interval height velocity (partial correlation coefficient = 0.40, *P* = .0048) However, change in CXM did not correlate with change in height velocity or change in height SD during treatment
Hoover-Fong et al, 2023 [48]	Conference poster	Sample size: 20, age 6-14 years Mean treatment duration 76 ± 14 (maximum 97) months	Height *Z*-score 1.56 ± 0.63) at Month 72 and 1.46 ± 0.69 at month 84 relative to an untreated ACH population. Significant ↓ in the upper body-to-lower body ratio No new safety issues; no drug-related serious AEs
Savarirayan et al, 2021 [49]	Report of an extension trial (NCT03424018) of NCT03197766	2-year results from an open-label, phase 3 extension study Sample size: 119 All participants received vosoritide at a dose of 15.0 μg/kg/day	In children randomized to vosoritide (*n* = 43), annualized growth velocity increased from 4.26 cm/year at baseline to 5.39 cm/year at 52 weeks and 5.52 cm/year at week 104 In children who crossed over from placebo to vosoritide in the extension study (*n* = 44), annualized growth velocity increased from 3.81 cm/year at week 52 to 5.43 cm/year at week 104 No new AEs of vosoritide were detected
Savarirayan et al, 2024 [50]	Report of an extension trial (NCT03424018) of NCT03197766	Sample size: 119, age 9.7 ± 2.6 years Mean treatment duration 4 ± 0.78 years	At year 3, the largest mean (SD) changes were observed in the Quality of Life of Short Stature Youth physical score (5.99 [19.41], caregiver reported; 6.32 [20.15], self-reported) and social score (2.85 [8.29] and 6.76 [22.64], respectively) Maximum benefit was observed in children with a more pronounced change in height z-score
Savarirayan et al, 2025 [51]	Report of an extension trial (NCT03424018) of NCT03197766	6-year results from an open-label, phase 3 extension study Sample size: 111 All participants received vosoritide at a dose of 15.0 μg/kg/day	Three-year comparisons of treated vs untreated children demonstrated an additional height gain of 5.75 cm (95% CI 4.93-6.57) with vosoritide A significant improvement in upper-to-lower body segment ratio at 3 years of treatment was observed for participants with assessments at age <11 (females) and <12 years (males) vs population-level, age-matched, untreated controls (*P* = .0087) The arm span-to-standing height ratio remained consistent with untreated participants •Vosoritide had a favorable safety profile with continuous treatment for up to 6 years; no long-term harms or deaths were observed

Abbreviations: ACH, achondroplasia; AE, adverse event; AUC, area under the curve; BP, blood pressure; cGMP, cyclic guanosine monophosphate; CXM, collagen X biomarker; PD, pharmacodynamic; PK, pharmacokinetic; ISR, injection site reactions; IVTLS, integrated vosoritide therapy with limb surgery; s.c., subcutaneous.

### Risk of bias in the included studies

**Figure 2:**
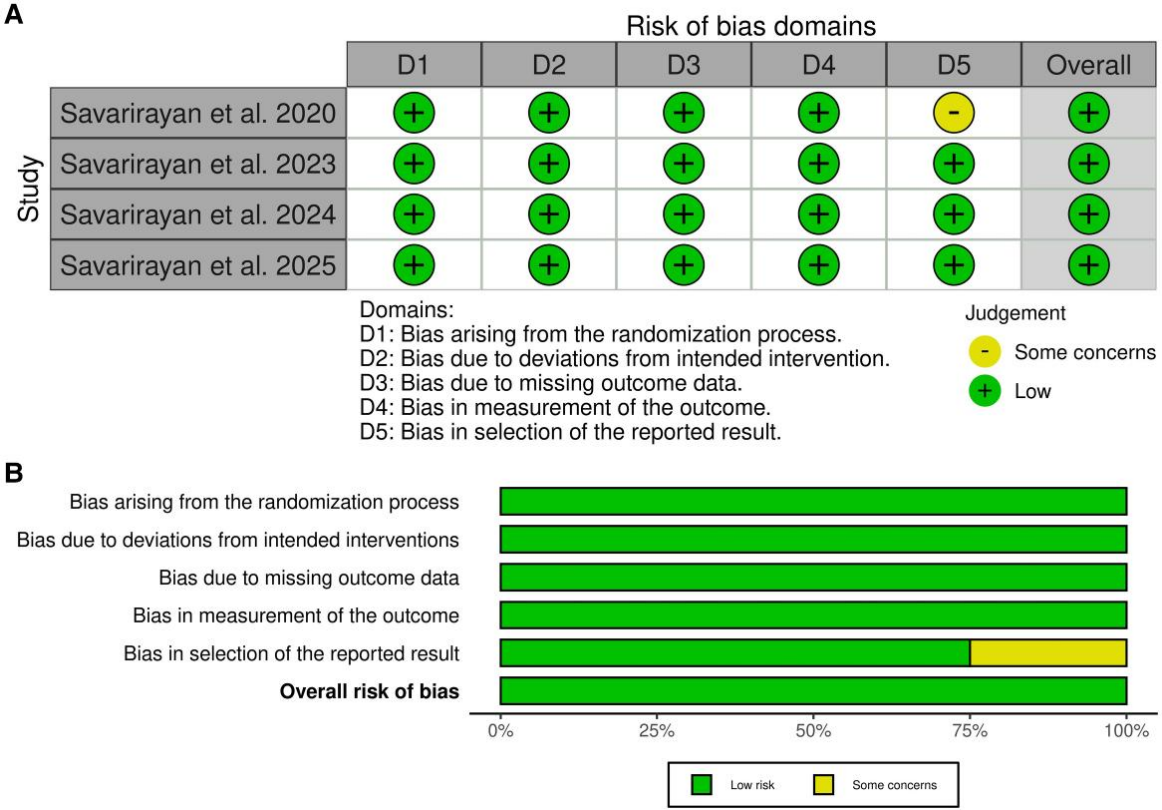
(A) Risk of bias summary: Review authors’ judgments about each risk of bias item for each included randomized controlled trial using RoB2. (B) Risk of bias graph: Review authors’ judgments about each risk of bias item presented as percentages across all included randomized controlled trials.

**Figure 3:**
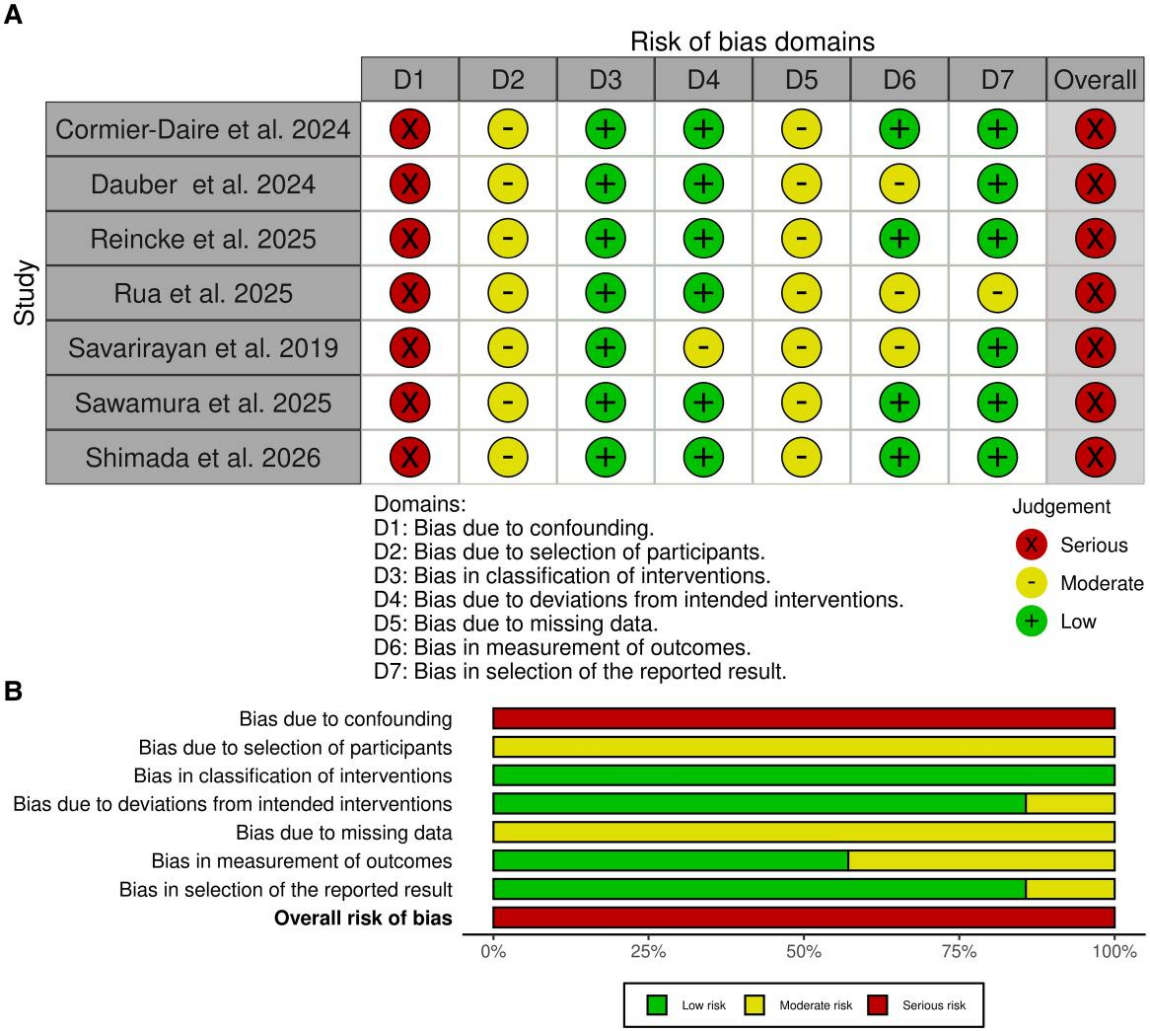
(A) Risk of bias summary: Review authors’ judgments about each risk of bias item for each included nonrandomized study using ROBINS-I. (B) Risk of bias graph: Review authors’ judgments about each risk of bias item presented as percentages across all included studies.

Figure 2 illustrates the specific and overall RoB in the included RCTs using RoB2. All RCTs showed low risks of overall bias, although 1 study (Savarirayan et al [26]) raised some concerns about bias related to the selection of reported results. All included nonrandomized studies demonstrated a serious risk of overall bias due to confounding bias, as assessed with ROBINS-I (Fig. 3). Additionally, these studies exhibited a moderate risk of bias related to participant selection and missing data. Publication bias was not assessed because the forest plots included fewer than 10 studies [52].

### Safety outcomes

Table 5 summarizes the comparison of safety outcomes between the CNP analogs and placebo groups. Compared with placebo, CNP analogs did not increase the risk of any AEs, treatment-related AEs, or serious AEs. CNP analogs were associated with higher risks of injection site reaction (RR 1.65; 95% CI [1.29-2.10]; *P* < .0001; *I*^2^ = 0%), urticaria (RR 4.04; 95% CI [1.18-13.86]; *P* = .03; *I*^2^ = 0%), and swelling (RR 3.57; 95% CI [1.77-7.23]; *P* = .0004; *I*^2^ = 0%) compared with placebo, but did not increase the risk of injection site erythema. Both groups had identical risks of rash, decreased blood pressure, pyrexia, nasopharyngitis, upper respiratory tract infection, influenza, nasal congestion, rhinorrhea, cough, oropharyngeal pain, ear pain, ear infection, otitis media, diarrhea, vomiting, gastroenteritis, conjunctivitis, headache, arthralgia, fractures, pain in the extremity, sleep apnea syndrome, and adenoidal hypertrophy.

**Table 5:** Comparison of the safety outcomes in the CNPA vs the placebo arms

Outcome variables	No. of study reports	No. of participants with outcome/participants analyzed		Pooled effect size, RR (95% CI)	*P*	*I* ^2^ (%)
		CNPA arm	Placebo arm			
Any AEs	4	183/191	132/135	1.00 (0.96-1.03)	.80	0
Treatment-related AE	3	75/159	63/103	1.04 (0.90-1.19)	.60	0
Serious AEs	4	11/191	13/135	0.61 (0.28-1.35)	.22	0
Injection site reaction	4	85/191	47/135	1.65 (1.29-2.10)	<.0001	0
Injection site erythema	3	67/134	54/108	1.30 (0.73-2.29)	.37	67
Injection site urticaria	2	12/92	3/93	4.04 (1.18-13.86)	.03	0
Injection site swelling	3	31/134	8/108	3.57 (1.77-7.23)	.0004	0
Rash	3	6/134	6/108	0.85 (0.29-2.50)	.77	0
BP decreased	4	8/191	6/135	0.82 (0.16-4.37)	.82	45
Pyrexia	4	53/191	43/135	0.84 (0.60-1.18)	.31	0
Nasopharyngitis	4	49/191	38/135	0.90 (0.62-1.30)	.56	0
Upper RTI	4	40/191	26/135	1.12 (0.71-1.76)	.63	0
Influenza	3	10/134	6/108	1.45 (0.56-3.79)	.44	0
Nasal congestion	3	14/134	13/108	0.78 (0.38-1.58)	.49	0
Rhinorrhea	2	12/74	7/47	1.17 (0.47-2.90)	.74	0
Cough	3	23/134	18/108	0.89 (0.47-1.66)	.71	0
Oropharyngeal pain	3	11/134	9/108	0.95 (0.40-2.24)	.91	0
Ear pain	3	11/134	8/108	1.13 (0.43-2.97)	.81	6
Ear infection	3	12/134	13/108	0.86 (0.41-1.79)	.89	0
Otitis media	4	29/191	22/135	0.84 (0.50-1.40)	.50	0
Diarrhea	3	17/134	11/108	1.18 (0.58-2.40)	.65	0
Vomiting	4	40/191	35/135	0.88 (0.39-1.97)	.75	66
Gastroenteritis	3	12/134	10/108	0.86 (0.36-2.05)	.74	0
Conjunctivitis	3	7/134	8/108	0.87 (0.35-2.19)	.77	0
Headache	4	30/191	25/135	0.94 (0.57-1.57)	.82	0
Arthralgia	3	15/134	5/108	2.23 (0.87-5.71)	.10	0
Fractures	3	2/149	1/120	1.60 (0.20-12.72)	.65	0
Pain in extremity	3	16/134	6/108	1.81 (0.69-4.73)	.23	0
Sleep apnea syndrome	4	4/191	1/135	1.33 (0.28-6.35)	.72	0
Adenoidal hypertrophy	2	1/92	2/93	0.63 (0.08-5.01)	.66	0

Abbreviations: AE, adverse event; BP, blood pressure; CNPA, C-type natriuretic peptide analogs; RR, risk ratio; RTI, respiratory tract infection.

In the single-arm trials and real-world studies, serious AEs and treatment-related discontinuations occurred infrequently. The most common AEs reported included injection site reactions, pyrexia, cough, gastrointestinal AEs, headache, and hypotension, as summarized in Table 5.

### Annualized growth velocity

**Figure 4:**
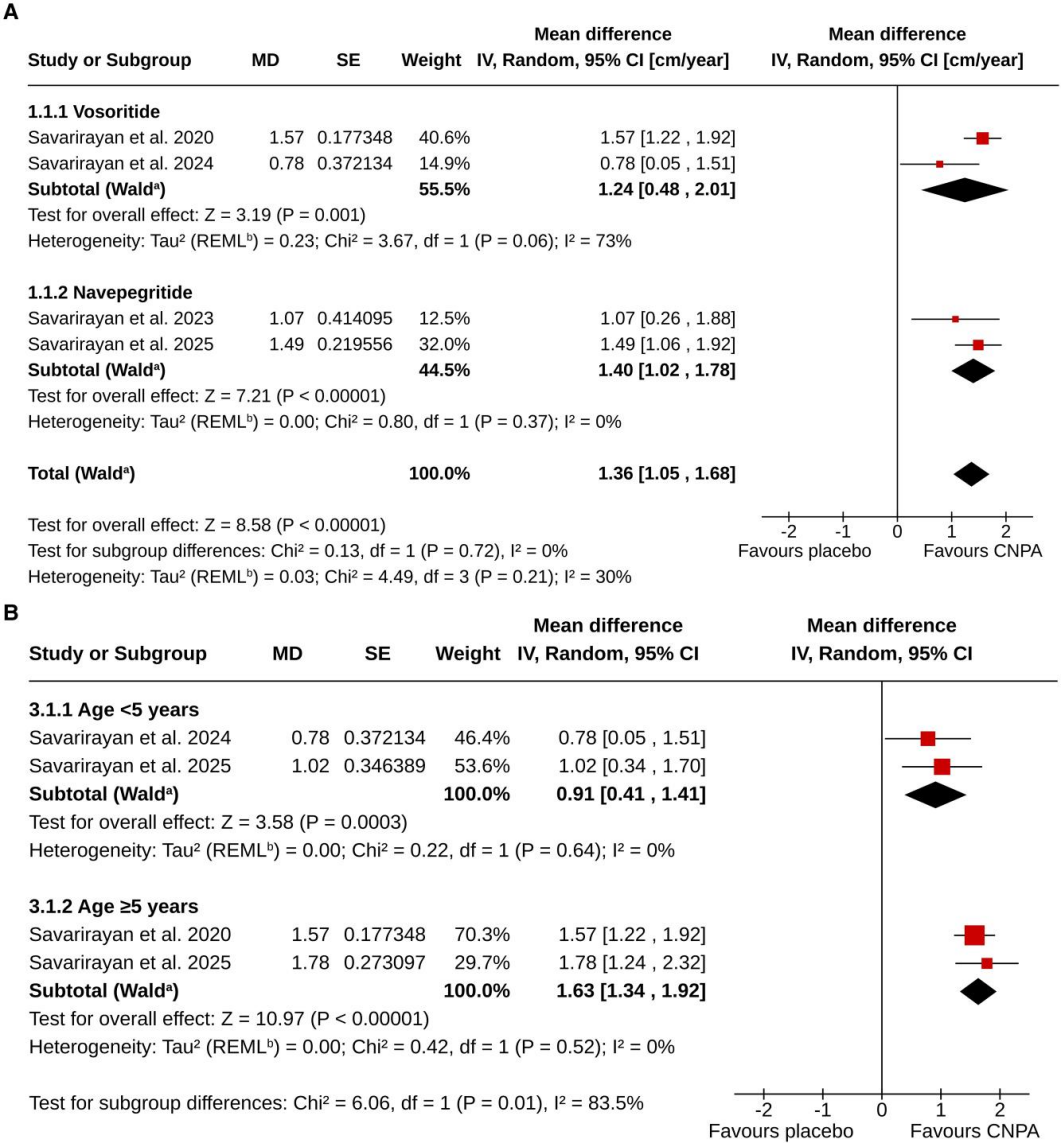
Forest plots depicting the change in annualized growth velocity from baseline to the end of the randomized controlled trials in the CNP analog vs placebo groups: (A) vosoritide and navepegritide subgroup; (B) in subgroups of patients with an age of <5 years and age ≥5 years at enrollment.

At the end of the trials, the use of CNP analog was associated with a greater increase in AGV from baseline than placebo (MD 1.36 cm/year; 95% CI [1.05-1.68]; *P* < .00001; *I*^2^ = 30%). This benefit was observed with both vosoritide (MD 1.24 cm/year; 95% CI [0.48-2.01]; *P* = .001; *I*^2^ = 73%) and navepegritide (MD 1.40 cm/year; 95% CI [1.02-1.78]; *P* < .00001; *I*^2^ = 0%), with comparable effects of the 2 drugs on AGV (*P* for subgroup differences = 0.72) (Fig. 4A). In subgroup analysis, such an increase in AGV was more pronounced in patients aged ≥5 years (MD 1.63 cm/year; 95% CI [1.34-1.92]; *P* < .00001; *I*^2^ = 0%) than in those aged <5 years (MD 0.91 cm/year; 95% CI [0.41-1.41]; *P* = .0003; *I*^2^ = 0%) (*P* for subgroup differences = .01) (Fig. 4B).

The noncontrolled studies reported mean changes in AGV ranging from 1.1 to 1.81 cm/year (Table 6). Reincke et al [40] found that children aged ≥10 to <16 years exhibited the highest mean AGV and AGV *Z*-score. Dauber et al [39] reported a significant increase in AGV SDS of 2.26 cm/year; in addition, they reported that the increment in AGV was significant for participants aged 3 to <5 and 5 to <9 years, but not for 9 to <11 years. Shimada et al [44] reported that ACH-specific AGV-SDS remained stable over the observation period in Group A and showed no significant changes in Group B.

**Table 6:** Reported efficacy and safety outcomes of the nonplacebo-controlled trials and real-world observational studies

Study	AEs	Reported height outcomes (mean ± SD or mean [CI])
Cormier-Daire et al, 2024 [38]	Total 21 AEs in 57 subjects, ISR 14, vomiting 3 All AEs were mild, no serious AEs No treatment-related discontinuations	At 12 months: AGV: 6.0 ± 1.44 cm/year Δ CDC height *Z*-score: 0.38 ± 0.33 Δ ACH height *Z*-score: 0.39 ± 0.26 Δ Absolute height: 6.2 ± 1.5 cm
Dauber et al, 2024 [39]	ISR (83.3%), fever (54.2%), cough (50%), vomiting (45.8%), rhinorrhea (25%), headache (25%), abdominal pain (20.8%) No treatment-related discontinuations One serious AE	At 12 months: AGV: 6.93 ± 0.93 cm/year; Δ 1.81 (1.16-2.46), *P* < .0001 AGV SDS: 1.12 ± 1.05 cm/year; Δ 2.26 (1.48-3.05), *P* < .0001 Height SDS: −2.91 ± 0.68; Δ 0.36 (0.26-0.47), *P* < .0001 ACH-specific height SDS: 0.03 ± 0.82; Δ 0.38 (0.20-0.55), *P* < .0001 Increase in AGV was significant for participants aged 3 to <5 years, and 5 to <9 years, but not 9 to <11 years
Reincke et al, 2025 [40]	12 of the 34 patients reported mild local ISR Two experienced dizziness as a moderate AE	At 12 months: AGV: *Age ≥2 to <5 years:* 6.27 ± 1.68 cm/year *Age ≥5 to <10 years:* 5.77 ± 1.24 cm/year *Age ≥10 to <16 years:* 6.98 ± 1.44 cm/year AGV *Z*-score: *Age ≥2 to <5 years:* 1.77 ± 1.36 (*P* = .0434) *Age ≥5 to <10 years*: 2.14 ± 1.32 (*P* < .0001) *Age ≥10 to <16 years:* 2.30 ± 1.23 (*P* = .0005) CDC height *Z*-score: −4.46 ± 1.06; Δ 0.38 ± 0.44, *P* < .0001 ACH height *Z*-score: 0.89 ± 1.37; Δ 0.52 ± 0.35, *P* < .0001 No significant changes in head circumference or body proportions (sitting height/height)
Rua et al, 2025 [41]	AEs in 66.7% subjects: ISR (51.9%), hypertrichosis (14.8%) No serious AEs No treatment-related discontinuations	At 24 months: AGV: 5.87 (5.13-6.60) cm/year; Δ 1.62, *P* ≤ .0001 WHO height SDS: Δ + 0.56 SD (*P* ≤ .0001) ACH height SDS: Δ + 0.95 SD (*P* ≤ .0001) ACH arm span SDS: Δ + 0.32 SD (*P* ≤ .01) ACH sitting height SDS: Δ + 0.79 SD (*P* ≤ .01) ULS ratio: Δ −0.10 (*P* ≤ .01) Most treated girls surpassed the 50th percentile on the achondroplasia-specific reference charts, while several boys reached or exceeded the 97th percentile
Savarirayan et al, 2019 [42]	AEs in 100% subjects: ISR (86%), pyrexia (54%), cough (49%), hypotension (46%), headache (40%) Serious AEs in 11% 17% treatment-related discontinuations	At 42 months: AGV: *Vosoritide 15 μg/kg/day:* 5.51 cm/year (between 30 and 42 months), Δ 1.46 (−0.15 to 3.07) *Vosoritide 30 μg/kg/day:* 5.6 cm/year (between 18 and 30 months), Δ 1.1 (−0.27 to 2.48) Δ CDC height *Z*-score: Overall 0.87 ± 0.696 (*Cohort 1:* 0.98 ± 0.99; *Cohort 2:* 0.49 ± 0.49: *Cohort 3:* 1.03 ± 0.57; *Cohort 4*: 1.06 ± 0.30) Δ ULS ratio: Overall −0.09 ± 0.121 (*Cohort 1:* −0.15 ± 0.079; *Cohort 2:* −0.13 ± 0.106: *Cohort 3:* −0.03 ± 0.132; *Cohort 4*: −0.13 ± 0.066)
Sawamura et al, 2025 [43]	Weight gain in 1 patient and postinjection mood disorder in another No treatment-related discontinuations	At 12 months: Height: Δ 5.4 ± 1.3 cm ACH height SDS: Δ 0.3 ± 0.2 (*P* < .01) Decreased the exaggerated lumbar lordosis and improved genu varum deformity
Shimada et al, 2026 [44]	Injection site pain occurred in 13.6% 4.5% treatment-related discontinuations	ACH-specific height SDS: *Group A (at 6 years)*: Δ 0.83 ± 0.42, *P* = .0013 *Group B (at 2.5 years):* Δ 2.15 ± 0.56, *P* = .0054 ACH-specific AGV SDS: *Group A (at 6 years)*: Δ 0.95 ± 2.45, *P* = NR *Group B (at 2 years):* Δ 2.13 ± 1.61, *P* = NR Greater benefit when started at ≥4 years, though earlier use may offer advantages

Abbreviations: ACH, achondroplasia; AE, adverse event; AGV, annualized growth velocity; CDC, US Centers for Disease Control and Prevention; CNPA, C-type natriuretic peptide analogs; ISR, injection site reactions; NR, not reported; SDS, SD scores; WHO, World Health Organization.

### Secondary outcomes

**Figure 5:**
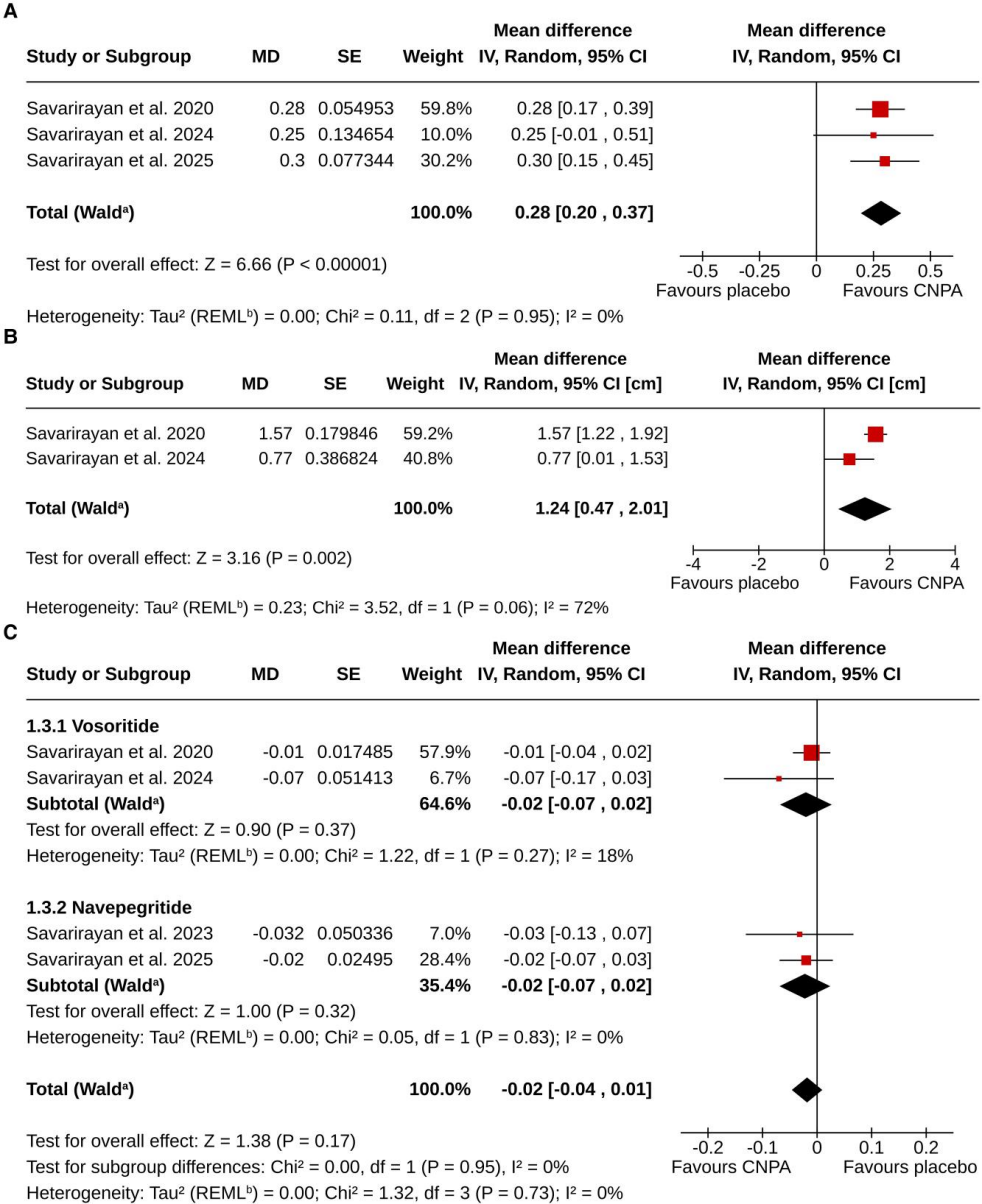
Forest plots depicting the change in the secondary height outcomes from baseline to the end of the randomized controlled trials in the CNP analog vs placebo groups: (A) height *Z*-score; (B) standing height; and (C) upper-to-lower segment body ratio.

At the end of the trials, CNP analogs significantly improved height *Z*-score (MD 0.28; 95% CI [0.20-0.37]; *P* < .00001; *I*^2^ = 0%) (Fig. 5A) and standing height (MD 1.24 cm; 95% CI [0.47-2.01]; *P* = .002; *I*^2^ = 72%) (Fig. 5B) compared with placebo. However, they had no effect on the ULS ratio (MD −0.02; 95% CI [−0.04-0.01]; *P* = .17; *I*^2^ = 0%), with similar results for vosoritide (MD −0.02; 95% CI [−0.07-0.02]; *P* = .37; *I*^2^ = 18%) and navepegritide (MD −0.02; 95% CI [−0.07-0.02]; *P* = .32; *I*^2^ = 0%) (Fig. 5C).

The noncontrolled studies showed an increase in height *Z*-score from 0.38 to 0.87, and in ACH-specific chart height *Z*-score from 0.39 to 0.52. In these studies, height SDS (US Centers for Disease Control and Prevention or World Health Organization) increased by 0.36 to 0.56, whereas ACH-specific height SDS increased by 0.3 to 0.95. There was a 5.4- to 6.2-cm gain in standing height. Rua et al [41] and Savarirayan et al [42] reported reductions in the ULS ratio of −0.10 and −0.09, respectively. Reincke et al [40] reported no significant changes in head circumference or body proportions (sitting height-to-standing height). Reduction in exaggerated lumbar lordosis and improvement in genu varum were observed in Sawamura et al's study [43]. The height outcomes reported by the nonplacebo-controlled trials and real-world observational studies are summarized in Table 6.

### Other outcomes

Savarirayan et al [28] investigated changes in skull and brain morphology, including the foramen magnum, as well as ventricular and brain parenchymal dimensions. They found that, in cohort 3, facial volume increased by 44% vs 34%, sinus volume increased by 129% vs 48%, and the foramen magnum area increased by 44% vs 25% in the vosoritide and placebo groups, respectively. Among older children in cohorts 1 and 2, these changes were less pronounced, with no noticeable difference between the 2 groups. Savarirayan et al [29] demonstrated that improvements in lower-limb alignment were observed with navepegritide compared with placebo, as shown by reductions in the tibial-femoral angle, mechanical axis deviation, and fibula-to-tibia length ratio. They also reported numerical improvements across all subscales of the Achondroplasia Child Experience Measures–Physical Functioning in the navepegritide group vs the placebo group [28]. The RCTs found no meaningful difference in quality-of-life changes (measured by tools such as Pediatric Quality of Life Inventory, SF-10 Health Survey for Children, Sheehan Disability Scale, the Quality of Life Short Stature Youth, and Parent Global Impression Items) between the placebo and CNP groups [26-28].

## Discussion

This systematic review demonstrates that C-type natriuretic peptide (CNP) analogs confer a slight increase in linear growth in children with ACH, with AEs largely confined to local injection reactions and transient blood pressure changes, and no clear signal of serious systemic harm observed over short- to mid-term follow-up. However, key uncertainties remain regarding optimal timing of treatment initiation, long-term skeletal benefits, and ultimate effects on adult height and function, and long-term complications.

### Efficacy and mechanistic plausibility

Across the RCTs with low overall RoB involving 326 participants [19, 26-28], CNP analogs increased AGV by approximately 1.4 cm/year and slightly improved height SDS over 52 weeks, with broadly similar effects observed for vosoritide and navepegritide. Direct head-to-head comparisons are unavailable, and indirect comparisons are inappropriate given heterogeneity in study design, populations, and endpoints.

Subgroup analyses suggest important age-related nuances. Vosoritide demonstrated consistent AGV improvement across all studied age groups (5-17 years), indicating broadly uniform responsiveness once treatment is initiated after early childhood. In contrast, navepegritide showed more pronounced age-dependent effects in children aged 2-11 years, with larger gains observed in older children. These differences should be interpreted cautiously, as the pivotal vosoritide trials excluded children younger than 5 years, limiting age-matched comparisons in early childhood [29, 42]. We observed notable heterogeneity within the vosoritide subgroup regarding growth velocity. This divergence between the Savarirayan et al 2020 and 2024 datasets likely reflects age-related differences in growth dynamics. The 2024 data include younger cohorts (infants/toddlers), where natural growth velocity is higher and more variable, and measurement techniques (recumbent length vs standing height) differ. In contrast, the navepegritide subgroup showed zero heterogeneity, potentially due to more homogenous inclusion criteria regarding age or Tanner stage in those specific trial phases. Japanese real-world data showed that children starting vosoritide at ≥4 years demonstrated significant improvements in ACH-specific height SDS, while those starting before age 4 maintained stable scores [44]. Future analyses should stratify strictly by age (eg, <5 vs 5-11 vs >11 years) and take into account pubertal staging to isolate these effects. Higher growth rate observed among patients between 10 and 16 years could be related to the additive effects of endogenous sex hormones in this age group.

The observed gains in AGV are mechanistically consistent with the molecular pathophysiology of ACH. CNP acts downstream of *FGFR3*, antagonizing *FGFR3*-mediated *MAPK/ERK* signaling and partially restoring growth plate chondrocyte function. When applied to ACH-specific growth curves, the treatment-associated gains narrow but do not close the height gap relative to average-stature peers, supporting the concept of partial disease modification rather than complete normalization in the short to medium term [41]. It is important to note that AGV is an extrapolated metric (projecting the rate over a full year), whereas the standing height data represent the actual, measured “extra” height achieved during the study. Because CNP analogs act at the NPR2–cGMP–protein kinase G (NPR2–cGMP–PKG) node rather than at the receptor level, their effects are expected to amplify chondrocyte proliferation without fully correcting upstream spatial patterning cues. The incremental changes in height and AGV, without short-term change in upper-to-lower segment ratio, are congruent with this biology, suggesting that stature can improve while long-standing skeletal disproportionality persists. Notably, the vosoritide extension study reported a significant improvement in upper-to-lower segment ratio at 3 years [51], although further long-term data are required to substantiate this observation. Achondroplasia is characterized by rhizomelic shortening; for the ratio to normalize, the proximal leg length must increase disproportionately faster than distal leg and trunk length. The lack of statistical significance here suggests that while CNP analogs increase overall linear growth, they may promote growth in the spine and extremities at relatively similar rates in the short term. It remains to be seen whether earlier initiation of treatment (eg, in infancy) or longer durations of therapy will eventually improve proportionality, which is critical for functional outcomes such as reach and gait mechanics.

Extension and real-world datasets indicate that multiyear vosoritide exposure yields cumulative height gains of several centimeters compared with natural history cohorts, with arm span-to-height ratios remaining close to those of untreated controls and no evidence of generalized overgrowth. A recent systematic review and meta-analysis synthesized multinational real-world cohorts of children with genetically confirmed ACH treated with vosoritide in routine care or early-access programs [53]. Across observational studies with ≥6 to 12 months’ follow-up, vosoritide increased AGV by approximately 1.5 to 2.0 cm/year over pretreatment or untreated controls, with mean on-treatment velocities around 6 cm/year. Height SDS improved vs ACH-specific references (≈+0.3 to +1.0 SD over 1-2 years) [53]. It is yet to be seen in long-term studies as to whether there are alterations in body proportion that are significant enough to prevent complications driven by canal diameter or pelvic and limb geometry.

Navepegritide demonstrated early improvements in skeletal alignment at 52 weeks, including normalization of fibular-to-tibial growth ratios and reductions in lower-limb angular deformities, without acceleration of bone age or adverse effects on spinal curvature [29]. While longer-term data are pending, these findings raise the possibility that CNP analogs, especially longer-acting formulations, may influence biomechanical stress and downstream orthopedic outcomes in addition to linear growth.

### Outstanding knowledge gaps

Substantial knowledge gaps persist across the lifespan, including the optimal age to initiate and discontinue therapy, the impact on skeletal architecture and downstream complications, and long-term effects on mortality, morbidity, and quality of life. While most RCTs initiated treatment in children with open epiphyses to maximize growth potential, the ideal timing of treatment initiation has not been defined.

Currently, there are no data demonstrating whether early initiation alters the natural history of foramen magnum stenosis, spinal canal narrowing, or need for neurosurgical intervention compared with historical cohorts. Available follow-up over 4-6 years shows sustained gains in growth, stable proportional indices, and no emerging structural toxicities, but does not clarify whether treated individuals experience fewer lower-limb deformities, less back pain, or reduced requirements for spinal and limb surgery in adulthood [51]. In infants and toddlers, foramen magnum stenosis is a major driver of early mortality, yet the effect of CNP analogs on this critical component remains essentially unknown.

In the phase 2 vosoritide RCT, cranio-cervical MRI assessments were limited to morphometric measurements, and the study was neither designed nor powered to detect changes in clinical outcomes such as progression of cord indentation or brainstem cerebrospinal fluid effacement over 52 weeks. Consequently, current evidence cannot determine whether early vosoritide treatment modifies the natural history of skull base stenosis or reduces lifetime need for decompression, underscoring the need for future trials incorporating standardized neuroimaging and neurological endpoints. Other unknown aspects are whether there are beneficial effects on sleep-disordered breathing, spinal canal stenosis, and reduced need for surgical interventions. Preventing neurological complications and quality of life may ultimately require strategies beyond height gain alone, potentially including earlier, longer, or combination approaches.

Children enrolled in vosoritide studies will be followed until they achieve final adult height to assess durability of response and whether a pubertal growth velocity increase is restored (normally attenuated in ACH) [26, 54]. Studies are underway to assess vosoritide use in children aged 0 to <5 years to evaluate whether earlier treatment leads to better outcomes in final height, body segment proportionality, and neurosurgical and orthopedic comorbidities.

Evidence in skeletally mature individuals is virtually absent, and existing data do not support use of CNP analogs for established adult complications dominated by canal narrowing, joint degeneration, and chronic pain. Whether childhood treatment translates into improved adult physical function, employment, or cardiometabolic outcomes compared with natural history remains a central unanswered question for families contemplating years of daily or weekly injectable therapy.

### Safety profile

The pooled safety profile is reassuring over trial durations, with overall AEs, treatment-related events, and serious events occurring at rates similar to placebo, alongside expected increases in injection site reactions, urticaria, and local swelling. Vosoritide administration has been associated with mild, transient, and clinically inconsequential reductions in blood pressure that resolve spontaneously [26, 28], whereas hypotension has not been a prominent feature with navapegritide, likely reflecting its steady-release formulation and lower peak exposure [27, 29]. Longitudinal vosoritide data up to 6 years suggest a favorable safety profile without emergent major toxicities [51].

The tolerability difference between these agents is clinically meaningful, as the burden of daily injections with associated pain and anxiety represents a significant treatment barrier, especially in the pediatric population (personal clinical experience of author A.P.A.). Treatment discontinuations were uncommon and primarily related to injection burden rather than safety [26]. Navepegritide demonstrated fewer and milder injection site reactions and high adherence rates, highlighting the clinical relevance of dosing frequency and tolerability, especially in pediatric populations [29].

Preclinical studies and early human experience have not identified signals of pathologic overgrowth, but the underlying biology mandates ongoing pharmacovigilance. Chronic activation of a growth plate and vascular-modulating pathway in young children raises legitimate concerns regarding subtle regional overgrowth; long-term extension studies and disease registries will therefore be essential to exclude small but clinically important excess growth risks.

### Future therapeutic directions

The therapeutic landscape for ACH has broadened considerably in recent years. Importantly, this expanding paradigm is also increasingly relevant to related FGFR3-mediated skeletal dysplasia such as hypochondroplasia, in which CNP analogs such as vosoritide have been tried and shown to have similar positive effect on growth [39]. Infigratinib, an oral FGFR1-3-selective tyrosine kinase inhibitor, is under investigation as a direct strategy to counteract FGFR3 overactivity in the PROPEL and PROPEL 2 trials [55]. Like vosoritide and navepegritide, infigratinib seeks to rebalance overactive *FGFR3* signaling within the growth plate, but does so through direct inhibition of receptor kinase activity rather than amplification of the counter-regulatory NPR2–cGMP pathway. Both CNP analogs and infigratinib target the same pathogenic axis of excessive *FGFR3* signaling that disrupts endochondral ossification, albeit at different nodes and with distinct pharmacologic profiles. Infigratinib may offer the practical advantages of oral dosing and potentially broader correction of FGFR3-driven skeletal patterning but carries a higher theoretical risk of off-target effects (eg, on phosphate handling, lens, or other FGFR-dependent tissues) and will require careful dose finding to avoid excessive pathway suppression.

Additional FGFR3-selective,agents, including TYRA-300, in early clinical development, has demonstrated promising effects in Fgfr3Y367C/+ ACH and hypochondroplasia mouse models, increasing chondrocyte proliferation and differentiation and improving long-bone length, skull morphology, foramen magnum size, and spinal dimensions. A phase 2 clinical trial (NCT06842355, BEACH301) is evaluating TYRA-300 in children aged 3-10 years with ACH, with outcomes including safety, pharmacokinetics, and growth velocity [56]. Until additional agents gain regulatory approval, thoughtful integration of vosoritide therapy with optimally timed orthopedic and neurosurgical interventions will remain central to progressing from first-generation disease modification toward truly lifespan-oriented care in ACH. Future studies should evaluate the rate of skeletal maturation with different agents compared with placebo. These studies should also assess how early institution of such investigational agents affect final adult height.

### Strengths and limitations of this review

This systematic review is the first to quantify the cumulative effect of CNP analogs on AGV in ACH, reinforcing their impact on linear growth within a mechanistically informed framework. Additional strengths include a prespecified focus on growth outcomes, integration of RCT and extension data where available, low overall RoB in the RCTs included, and detailed consideration of mechanistic plausibility and emerging alternative therapies.

However, the review is constrained by the small number of eligible studies, variability in primary endpoints, and heterogeneity in trial design and follow-up duration. The absence of robust data on ACH-related complications beyond stature such as otitis media, cardiopulmonary outcomes, neurological sequelae, and foramen magnum–related complications substantially limits inferences regarding broader clinical benefit. Long-term outcomes into adulthood are currently sparse, and serious RoB within individual real-world studies, particularly around selective reporting, and incomplete outcome data, must be acknowledged when interpreting results.

The RoB primarily driven by small sample sizes, open-label extension phases, and use of AGV rather than final adult height as the primary outcome, resulting in imprecision and indirectness for long-term benefit are some important issues. Additional concerns include heterogeneity in study design, age ranges, and outcome definitions, which preclude reliable cross-trial comparisons. While extension data suggest persistence of growth effects, certainty of evidence for long-term clinically meaningful outcomes remains limited pending completion of longitudinal follow-up. The cost implications of CNP analog therapy (eg, average cost of vosoritide treatment can be US$ 382 866 annually) were not assessed in these studies, an important issue for Mediclaim and in resource poor settings.

## Conclusions

Current CNP analog therapies appear to partially counteract dysregulated *FGFR3* signaling in ACH, achieving slight gains in linear growth with an acceptable short-term safety profile. Sustained longitudinal follow-up and postmarketing registries are essential to define the durability of response, long-term safety, and real-world functional outcomes across the lifespan. Given that many comorbidities in ACH arise as downstream consequences of impaired skeletal growth, it is plausible that timely correction of the underlying growth plate pathology will favorably influence these complications, although this has yet to be demonstrated. Future work should systematically evaluate the impact of CNP analogs and emerging FGFR3-directed therapies on neurological, cardiopulmonary, orthopedic, and quality-of-life outcomes, as well as on morbidity and mortality relative to contemporary natural history control cohorts.

## Data Availability

Original data generated and analyzed during this study are included in this published article or in the data repositories listed in References.

## Registration information

The meta-analysis was registered in PROSPERO, with registration number CRD420251248977. The review protocol summary can be accessed at the PROSPERO website.

## Abbreviations

BMI: body mass index
NA: not available
PICOS: The Population, Intervention, Comparison, Outcomes, and Study design
PRISMA: Preferred Reporting Items for Systematic Reviews and Meta-Analyses
RevMan: Review Manager
RCT: randomized controlled trial
RoB: risk of bias
ROBINS-I V2: The Risk Of Bias In Nonrandomized Studies of Interventions, Version 2 tool
SR/MA: systematic review and meta-analysis
